# The Role of Hydrogen Peroxide in Redox-Dependent Signaling: Homeostatic and Pathological Responses in Mammalian Cells

**DOI:** 10.3390/cells7100156

**Published:** 2018-10-04

**Authors:** Noemi Di Marzo, Elisa Chisci, Roberto Giovannoni

**Affiliations:** School of Medicine and Surgery, University of Milano-Bicocca, via Cadore 48, 20900 Monza, Italy; noemi.dimarzo91@gmail.com (N.D.M.); e.chisci4@gmail.com (E.C.)

**Keywords:** hydrogen peroxide, redox regulation, oxidative stress

## Abstract

Hydrogen peroxide (H_2_O_2_) is an important metabolite involved in most of the redox metabolism reactions and processes of the cells. H_2_O_2_ is recognized as one of the main molecules in the sensing, modulation and signaling of redox metabolism, and it is acting as a second messenger together with hydrogen sulfide (H_2_S) and nitric oxide (NO). These second messengers activate in turn a cascade of downstream proteins via specific oxidations leading to a metabolic response of the cell. This metabolic response can determine proliferation, survival or death of the cell depending on which downstream pathways (homeostatic, pathological, or protective) have been activated. The cells have several sources of H_2_O_2_ and cellular systems strictly control its concentration in different subcellular compartments. This review summarizes research on the role played by H_2_O_2_ in signaling pathways of eukaryotic cells and how this signaling leads to homeostatic or pathological responses.

## 1. Introduction

In biology and medicine, hydrogen peroxide (H_2_O_2_) is often used as anti-infective agent, for example to cleanse wounds, because of its strong oxidant abilities that can kill microorganisms and cells [1]. H_2_O_2_ has a central role in homeostatic metabolism, being the key molecule in the Third Principle of the Redox Code (“Redox sensing through activation/deactivation cycles of H_2_O_2_ production linked to the NAD and NADP systems to support spatiotemporal organization of key processes” [2]) of the living organisms and it plays relevant roles in the regulation of the cellular metabolism. The H_2_O_2_ is, in fact, recognized as one of the main molecules in sensing, modulation and signaling of redox metabolism, acting as one of the main messenger molecules [3]. H_2_O_2_ together with other second messengers (nitric oxide, NO and hydrogen sulfide, H_2_S) are enzymatically produced as soon as a signal is sensed by a receptor. The second messengers activate in turn a cascade of downstream proteins via specific oxidations leading to a metabolic response of the cell. We can refer to this process as redox signaling [4]. In this context, one of the main biological sources of H_2_O_2_ involves the breakdown, either spontaneous or catalytic, of superoxide anions (O^2−^), produced by partial reduction of oxygen during aerobic respiration or by several oxidases, rapidly converted in H_2_O_2_ by superoxide dismutases, which are located in the mitochondria, in the cytosol or in the extracellular space [5]. H_2_O_2_ diffuses across the cell membranes via aquaporin water channels (AQP) [6] transducing the redox signal from the location where it was generated to a target site. H_2_O_2_ activates several transcription factors in bacteria, lower eukaryotes, and mammalian cells [7] and this activation ultimately leads to a metabolic response of the cell to the original stimuli. This review summarizes research on the role played by H_2_O_2_ in signaling pathways of eukaryotic cells and how this signaling leads to homeostatic or pathological responses.

## 2. The Source of H_2_O_2_ at the Beginning of Redox Signaling

The redox-dependent signaling is connected to the signal transduction pathways that originate from the binding of intracellular or membrane receptors to their specific ligands [4,8]. This mechanism can lead to the activation of specific target proteins, including transcription factors, as a response to physiological oxidative stress. H_2_O_2_ is involved in Reactive Oxygen Species (ROS) intracellular signaling as a mediator of several physiological processes such as cell differentiation and proliferation, cellular metabolism, survival, and immune response. Thanks to these features, H_2_O_2_ with both reducing and oxidizing properties can be classified as a second messenger molecule for cell metabolism. Furthermore, the function as a signaling intermediate can be placed in both a physiological and pathological settings [9].

Several one- or two- electron reduction reactions can act as source of H_2_O_2_, with nicotinamide adenine dinucleotide phosphate (NADPH) oxidases (NOXs) and mitochondrial respiratory chain being the major enzymatic generators [3]. In fact, in aerobic organisms the sources of O_2_^−^ can be found both in the extracellular space and in several cellular compartments, depending on which enzymes are producing it: the NOXs in the plasma membrane or phagosomes, the complex I and III of the respiratory chain in the mitochondria or the cytochrome P450-monooxygenases in the endoplasmic reticulum (ER) [4,10]. In mammals, one of the major synthesis processes of H_2_O_2_ is represented by the enzymatic reaction catalyzed by the superoxide dismutase (SOD) enzymes: the dismutation of superoxide to H_2_O_2_ and oxygen: 2O_2_^•−^ + 2H^+^ → H_2_O_2_ + O_2_. The presence of three SOD isoforms maintains the homeostasis and coordinates several ROS signals between cellular compartments. These isoforms work with specific metal cofactors and they have a different subcellular localization: SOD1 (Cu/Zn-SOD) is located in the cytoplasm, SOD2 (Mn-SOD) is located within the mitochondria, whereas SOD3 (Cu/Zn-SOD) has an extracellular localization [11].

The production of ROS from multi-enzymatic complex of NOXs in the plasma membrane is then involved in the intra- and extra-cellular signaling [10,12]. The NOX family members are transmembrane proteins with similar features to phagocytic NOX-2: their most important function is to transfer electrons across membranes, reducing oxygen into superoxide [13,14]. The molecular oxygen (O_2_) produced by the NOX-2 complex can be released into the extracellular space where it is converted into H_2_O_2_ by SOD3 enzyme or it could influx through the chloride channels [10]. H_2_O_2_ can also cross the cellular membrane via simple diffusion through AQPs, which are integral membrane proteins with a double function: AQP3 and AQP8 can facilitate H_2_O_2_ transition through the cell membrane and are thus involved in different downstream signaling cascades [6,8,15,16].

## 3. H_2_O_2_ as a Molecular Mediator of Cellular Signaling

**Figure 1:**
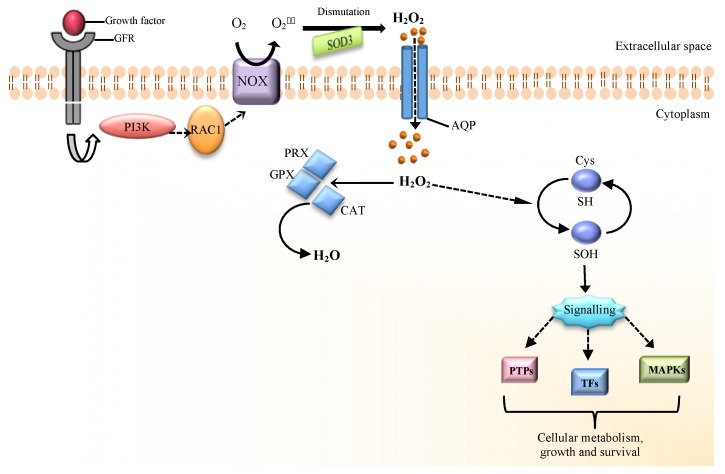
H_2_O_2_ signaling in mammalian cells. The binding of growth factors (e.g., EGF or PDGF) to their receptors triggers several downstream events. NADPH oxidase (NOX) is a membrane-bound enzyme complex that can produce superoxide anion (O_2_^•−^). Activation of this complex (e.g., NOX-2) occurs after the sequential activation of phosphatidylinositol-3-kinase (PI3K) and Rac small GTPase 1 (RAC1) proteins. O_2_^•−^ produced from NOX complex can dismutate to H_2_O_2_ by superoxide dismutase-3 (SOD3). H_2_O_2_ can cross the cellular membrane through aquaporin water channels (AQPs) and activates ROS signaling with oxidative modification of critical redox-sensitive Cys in signaling proteins. The targets of H_2_O_2_ include transcriptional factors (TFs), mitogen-activated protein kinases (MAPKs) and protein Tyr phosphatases (PTPs). Cellular antioxidant systems, such as catalase (CAT), glutathione peroxidases (GPXs) and peroxiredoxins (PRXs) cooperate to maintain redox homeostasis [9,10].

The most important and characterized mechanism by which the H_2_O_2_ molecule acts as a mediator of the cellular signaling is the reversible oxidation of specific cysteine (Cys) residues (Figure 1). This process mainly involves those redox-sensitive proteins which have metabolic regulatory functions [17]. The burst of ROS generation is triggered from an increase in tyrosine (Tyr) phosphorylation, which typically occurs when growth factors (e.g., EGF or PDGF) bind to their receptors (e.g., EGFR, PDGFR). In this setting, the main targets involved in the redox regulation are the protein Tyr phosphatases (PTPs) that, together with the protein tyrosine kinases (PTKs), maintain homeostatic Tyr phosphorylation status to regulate signaling events in response to cytokines and growth factors mediated stimuli [18,19]. The enzymatic activity of PTPs is relative on the conserved reactive Cys residue of their active site. All PTPs proteins have an active site with essential Cys that exists as a thiolate anion (Cys-S^−^) susceptible to oxidation: if Cys are oxidized, then inactivation of PTP occurs. Usually, the PTPs inactivation is an event that can be reversed by glutathione (GSH), acting as a reducing agent, thus allowing the regulation of a signaling response [5,10,20,21]. Inactivation of PTP enzymes shifts the balance towards the activities of the Tyr kinase with a consequent raise in ligand-stimulated Tyr phosphorylation: concentrations of H_2_O_2_ to oxidize Cys residue must be elevated [9,10,11]. The thiol (RSH) groups of Cys have different redox states and, since their reactivity can be conditioned by the protein context, they allow selectivity in the redox signaling [10,22]. In particular, Cys are highly conserved especially since they are not abundant in proteins. One key factor in oxidation reactivity of Cys residues is low p*K_a_* values that are influenced by their local environment. Reactive Cys thiol groups exist as a thiolate anion (S^−^) under physiological pH. The first step of H_2_O_2_-mediated Cys oxidation lead to production of sulfonate or sulfenic acid (R-SOH), which is understood as a reversible oxidative state (sulfenylation) that leads to changes in the activity and conformation of a target protein. Since sulfenic acid is quite reactive, it is possible that another reaction is realized in the presence of nearby thiol to form a disulfide bond: if sulfenic acid reacts with a protein thiol or GSH, an inter/intramolecular disulfide bridge or protein-S-GSH disulfide can form, respectively [8,17,23]. The *floodgate* model is known as a mechanism able to induce the oxidation of the target proteins inactivating the scavenging enzymes by H_2_O_2_: local increases of H_2_O_2_ allow the inhibition of peroxiredoxins (PRXs) which follows the oxidation of a downstream target. The reversible inactivation of PRXs by sulfinic (SO_2_^−^) acid modification allows the buildup of endogenous H_2_O_2_ to promote signal transduction [23]. With high concentration levels of H_2_O_2_, due to R-SOH hyperoxidation, sulfinic (RSO_2_H), sulfonic (RSO_3_H) acids or their anions are produced: these irreversible modifications are representative of oxidative stress.

In addition to H_2_O_2_, the cells possess some other important second messenger molecules involved in the redox signaling. Several studies demonstrated that NO and H_2_S have a relevant role in the redox metabolism modulation both via a common pathway and single pathways. It has been reported that the NO/H_2_S common pathway can mediate the vasodilation, migration and proliferation of vascular cells and angiogenesis [24,25,26]. Moreover, H_2_S is involved in the upregulation of protective pathways that include vascular endothelial growth factor (VEGF), hypoxia-inducible factor 1-alpha (HIF-1α), and Phosphatidylinositol-4,5-bisphosphate 3-Kinase/AKT serine/threonine kinase (PI3K/AKT) [24,25,26]. In particular, H_2_S regulates the redox equilibrium that is important for cytoprotection and inhibition of oxidative stress [27]. In order to protect the cells from toxic effects induced by ROS under oxidative condition, the NO–H_2_S common pathway acts to inhibit mitochondrial complex I, cytochrome-*c* (cyt-*c*) component, and complex IV, which would otherwise favor the release of ROS. Furthermore, exogenous H_2_S stimulates NRF2 activation with a subsequent increase of anti-oxidant defense responses [24,27], as further detailed in Section 8. NO and H_2_S also regulate other enzymes such as PTPs through protein modifications. In particular, NO modifies PTP-1B by S-nitrosation at Cys 215 residue, which hinder inactivation by H_2_O_2_ induced irreversible oxidation [24,28]. H_2_S can react with free thiol decoupled to form a –SSH group through sulfhydration or it can react with dinitrogen trioxide (N_2_O_3_) derived from NO radical (NO^•^) and nitrite, to form nitrosothiol (-SNO). The latter is generated when NO reacts with a thiol: in the presence of GSH, -SNO can be modified into glutathionylated thiol (protein–SSG). When the sulfhydryl group of Cys is converted to an –SSH group, H_2_S can modifies Cys residues of the proteins: a modified Cys residue is highly reactive and leads to higher catalytic activity of targeted proteins [29,30]. For example, glyceraldehyde 3-phosphate dehydrogenase (GAPDH) can be modified both by S-sulfhydration and S-nitrosylation at Cys-150: NO inhibits the catalytic activity of GAPDH, while H_2_S increases it [31].

## 4. The H_2_O_2_ within the Cell: A Matter of Concentration

The concentration of H_2_O_2_ in the extracellular and intracellular space is of crucial importance for the metabolism and survival of the cells. Although the exact range may vary depending on cell type, the intracellular homeostatic concentration ranges from 1 to approximately 100 nM of H_2_O_2_: at higher concentrations stress or even inflammatory responses or growth arrest and cell death can be activated [3]. To maintain the homeostatic concentration of ROS, which depends on the balance between ROS formation and antioxidant systems, cells have developed defense mechanisms, both enzymatic and non-enzymatic. Enzyme defense systems against ROS include: the three isoforms of SOD (extracellular, cytosolic, and mitochondrial) whose dismutase activity leads to the production of H_2_O_2_ and water, as already mentioned in the previous paragraph; catalase (CAT), which is mainly present in the peroxisomes; glutathione peroxidases (GPXs); and PRXs. The CAT facilitates the disproportion of H_2_O_2_ into water (H_2_O) and molecular oxygen (O_2_) and it modulates its effects in two pathways based on the cellular concentrations of H_2_O_2_: low concentrations of H_2_O_2_ triggers the peroxidatic pathway promoting the oxidation of hydrogen donors such as phenols and alcohols. Conversely, in the presence of high concentrations of H_2_O_2_, the catalytic pathway (disproportion of H_2_O_2_) is favored [32]. The PRXs and GPXs families are located in different cell compartments: this different location is important to ensure that no increase or accumulation of H_2_O_2_ occurs in a certain cell compartment. GPX is a selenium-containing enzyme that operates catalytic reduction of H_2_O_2_ and lipid peroxides to water and lipid alcohols, respectively [5]. PRX and GPX families are involved in a mechanism of oxidation/reduction of Cys or selenocysteine residues. For instance, when H_2_O_2_ reacts with an oxidate PRX, the latter can convey this oxidation to the target protein, such as phosphatases or transcription factors [33]. Moreover, thioredoxin/thioredoxin reductase (Trx/TrxR) or GSH/glutathione reductase (GR) systems cooperate in order to make available the active and reduced form of enzymes (PRXs and GPXs) ensuring the reversibility of redox modifications derived from H_2_O_2_ [17]. The PRXs are subdivided into six members that have different amino acid sequences and at least one peroxidatic Cys that is present in the active site. These important scavenging enzymes are located in several cellular compartments such as the membrane, cytosol, mitochondria, endoplasmic reticulum, nucleus, and peroxisomes [8,33]. The other cell detoxification systems from ROS are the non-enzymatic ones, which include several molecules with antioxidant activity: vitamin A (β-carotene), vitamin E (α-tocopherol), vitamin C (ascorbic acid), urate, coenzyme Q, and GSH [34].

## 5. H_2_O_2_ and Apoptosis

**Figure 2:**
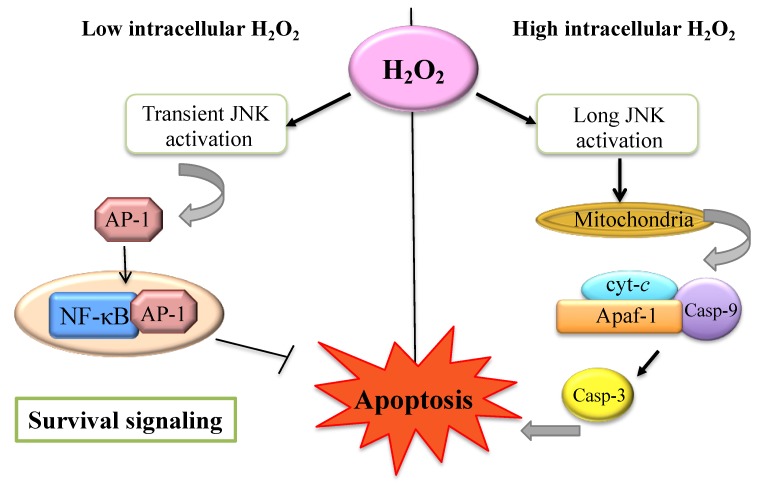
Survival and apoptotic signaling. High intracellular H_2_O_2_ induces long c-Jun NH2-terminal kinase (JNK) activation and lead to mitochondrial cyt-*c* complex release dependent cell death. Low intracellular H_2_O_2_ levels allow AP-1 transcription factor and anti-apoptotic genes activation [40].

As discussed in the previous paragraph, the cells exist in a redox equilibrium state between oxidative and reductive processes that occur continuously during the complex biochemical transformations of physiological metabolism. Disruptions in redox equilibrium manifest themselves when free radicals are formed in excess and/or these antioxidant substances are reduced or ineffective, leading to oxidative damage [35]. Concentrations of ROS higher than those tolerated by the cells and associated to the so-called oxidative distress [3] play a pivotal role in programmed cell death (PCD), as illustrated in Figure 2. The imbalance towards a pro-oxidant cellular state causes both cellular and subcellular (e.g., in the mitochondria) damage, cellular senescence, and cell death. Indeed, ROS generated within the mitochondria can feed back and directly damage mitochondrial DNA (mtDNA) and other mitochondrial components. The accumulation of oxidizing molecules causes the oxidation of DNA, lipids and proteins altering their role and their physical properties [35]. Among the roles H_2_O_2_ has in the regulation of cell growth, proliferation, and endothelial inflammatory responses, it also modulates endothelial apoptosis: H_2_O_2_ can be either detoxified by antioxidants or can generate a hydroxyl radical (OH^•^) from a Fenton reaction. OH^•^ causes damage to cellular proteins, lipids and nucleic acids, which, in turn, leads to apoptosis [36,37]. H_2_O_2_ can also induce autophagy cell death, which is a lysosomal degradation process [38]. The transient exposure to H_2_O_2_ triggers apoptosis via the mitochondrial pathway: the mitochondrial membrane hyperpolarization leads to the mitochondrial translocation of Bax and Bad (pro-apoptotic proteins that govern the permeability of the outer mitochondrial membrane) with cyt-*c* release from the mitochondria. The significant reduction of mitochondrial concentration of cyt-*c* further increases ROS production because of the breakdown of the electron transport chain. On the other hands, the significant increase of cyt-*c* concentration within the cytosol induces caspase-9-mediated activation of caspase-3 and the definitive execution of the apoptotic process [37,39].

The caspases belong to a highly conserved family of Cys-dependent aspartate proteases involved in apoptosis signaling pathways. In particular, caspase-2, despite being in the cytosol, possesses a nuclear localization signal (NLS) sequence allowing its transport into the nucleus; the caspase-2 activation depends on events related to oxidative stress such as ER stress, DNA damage and H_2_O_2_ exposure. It has been suggested that caspase-2 can be associated with the p53-inducible death domain-containing protein (PIDD) and the adaptor molecule RIP-associated ICH-1 homologous protein with a death domain (RAIDD) in order to engender the PIDDosome complex. The latter one is important for caspase-2 activation and processing in response to DNA damage: this pathway connects caspase-2 to p53-mediated cell death [37]. The pro-oxidative activity of p53 leads to the inhibition of the expression of antioxidant genes, causing an increase in oxidative cellular stress with consequent apoptosis. This inhibition mostly concerns the expression of SOD2 and NRF2, with consequent induction of apoptosis [41,42,43]. Furthermore, the upregulation induced by p53 of MnSOD and GPX increases oxidative stress and apoptosis, confirming that the balance between the antioxidant enzyme and oxidative stress is fundamental for cell survival [44,45]. Nevertheless, ROS-induced apoptosis requires the support of some cell death signaling pathways: the cellular components that most respond to oxidative stress generated by ROS are the members of the mitogen-activated protein (MAP) kinases family. In particular, the cellular pathways that respond to oxidative stress are the extracellular signal-regulated kinases (ERK1/2), c-Jun NH2-terminal kinases (JNKs), and p38 kinase. The ERKs family is involved in the response to growth factors (e.g., proliferation, differentiation) but can also be activated in response to ROS, such as superoxide and H_2_O_2_. Instead, the JNKs and p38 kinases are primarily involved in the cellular stress condition and are activated by H_2_O_2_ in smooth muscle cells [40,46,47,48].

## 6. H_2_O_2_, Bacteria, and Immune Cells

ROS are strictly controlled by antioxidant cell systems in order to prevent oxidative stress and avoid oxidative damage leading to cell death or protein dysfunction as discussed in the previous section: for this reason, the regulation of the cellular pro-oxidant/antioxidant systems is also important to facilitate resolution of an inflammatory state. One of the most important roles covered by H_2_O_2_ is to act as an anti-microbial agent. Cellular enzymes that have the function of producing H_2_O_2_ include the family of NOXs, which is composed of seven elements: NOX1-5 and the dual oxidases DUOX1 and DUOX2 [49,50,51]. The role of NOX has not been completely defined: NOX2 is known to be involved in the defense of the host, and the role of the other enzymatic members of this family may depend on the site of expression [52]. The interaction between bacteria and H_2_O_2_ is complex since it can lead to the production, by bacteria, of both H_2_O_2_ and enzymes that detoxify the latter such as CAT. The bacteria need a close interaction with the plasma membrane of the target cell to act on the host proteins and penetrate into the eukaryotic cells. Bacterial interaction with the apical cell membrane activates Toll-like receptors (TLRs) that stimulate the generation of extracellular H_2_O_2_ gradients: this mechanism leads to the production of hypothiocyanous acid (HOSCN) by myeloperoxidase (MPO) or lactoperoxidase (LPO) which catalyze the oxidation of thiocyanate (SCN^−^). Hypothiocyanous acid, a potent antimicrobial species, is correlated with the hypohalous acids (e.g., hypochlorous acid, HOCl, and hypobromous acid, HOBr) that are produced during the respiratory burst, defined as the high increase in oxygen demand and energy consumption at the cellular level, and together with other immune processes operate to stop the spread of pathogens [53]. DUOX-derived H_2_O_2_ gradients at the luminal surface remove bacteria from the epithelial surface and prevent the lesion, justified by the fact that the bacteria exhibit negative chemotaxis in H_2_O_2_ gradients [50,54]. The mechanism by which H_2_O_2_ allows a negative chemotaxis process may involve the activation of intracellular redox signaling (e.g., EGFR/ERK activation), the transcription nuclear factor (NF)-kB that regulates several genes involved in inflammation, as further discussed in Section 7 and Section 8, and the oxidative modifications of the Cys of the bacterial proteins responsible for the chemotactic process. Moreover, DUOX-derived H_2_O_2_ in epithelial wound responses acts as a guide for neutrophils by directing them towards the injured area, thus increasing the release of the neutrophil chemokine interleukin, IL-8 [50,55].

The cells that are mostly involved in the release of ROS during an inflammatory process or in tissue repair are the neutrophils, the macrophages/monocytes and the dendritic cells. Neutrophils are phagocytic white blood cells acting as the effectors of the innate immune system: they are recruited at the site of infection or inflammation to eliminate pathogens and foreign substances through the process of phagocytosis during which proteolytic enzymes and ROS are released. These cells maintain close contact with natural killer (NK) and dendritic cells (DCs), but also with key players in adaptive immunity, B cells and T cells [56,57]. T cells are often found close to phagocytic cells that release ROS during the respiratory burst: H_2_O_2_ is the most involved ROS in this mechanism. H_2_O_2_ is released by the NOX-2 enzyme system of activated phagocytes and can oxidize thiol groups on the surface of T cells. The dendritic cells and activated phagocytes release Cys into the extracellular space which is captured by the T cell and then converted into GSH. The latter keeps the thiol groups on the surface of the T cell in a reduced state and neutralizes the H_2_O_2_ inside the cell, allowing DNA synthesis. The T lymphocyte receptor (or TCR) is located on the surface of T lymphocytes and is responsible for the recognition of antigens presented by the major histocompatibility complex (MHC). The bond between TCR and the MHC-antigen complex leads to the activation of the T lymphocytes and the secretion of Trx from the T lymphocytes, DCs and regulatory T cells (Tregs). The main role of the Trx is to keep the thiols present on the surface in a reduced state [56].

## 7. H_2_O_2_ during Inflammation

H_2_O_2_ acts as a signal molecule and second messenger also in the inflammatory setting. The inflammatory response is characterized by different mediators and specific cell types that contribute to maintain tissue integrity and function. The innate immune system is made up of specialized cells that produce cytokines in response to various inflammatory stimuli: production of proinflammatory cytokines is an important requirement to allow the activation of adaptive immune system cells, such as T- and B-lymphocytes. As discussed in the previous section, inflamed tissues are associated with high levels of ROS, produced during the respiratory burst from immune cells playing an important role in antimicrobial host defense [49,50]. Several biologically active proinflammatory mediators stimulate the production of ROS by neutrophils, macrophages, and dendritic cells that reach the inflammation site to begin the tissue repair process and to prevent pathogens from attacking the damaged area. During inflammation and tissue injury ROS can interact with proteins, lipids, carbohydrates, nucleic acids, and other metabolites in order to moderate the inflammatory response ensuring cellular signaling [58]. During the inflammatory process, highly conserved damage signals are released, allowing the activation of important pathways that lead to transcription of genes involved in inflammation and wound response. These signals are ‘felt’ as potentially harmful (Damage Associated Molecular Patterns or DAMP) and are often components of the same pathogen, or they can be molecules that are released from the cells damaged by the event. The cell that receives these signals activates various types of receptors and subsequently downstream Nuclear Factor Kappa B (NF-κB), mitogen-activated protein kinase (MAPK), or type I interferon signaling pathways that are important for inflammatory and antimicrobial responses [49,50].

## 8. H_2_O_2_ and the Control of Gene Expression

**Figure 3:**
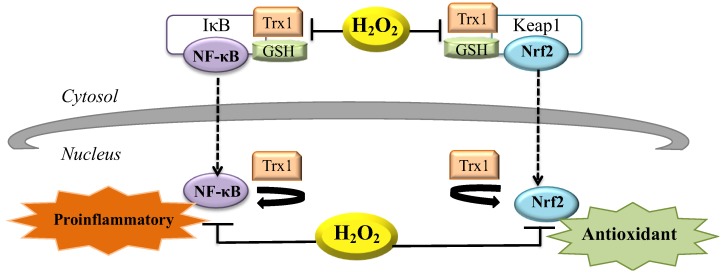
Proinflammatory and antioxidant signaling via H_2_O_2_. Release of inhibitory subunit (IκB) of NF-κB is due to oxidation by cytosolic H_2_O_2_ that leads to dissociation of the NF-κB/IκB complex. Once in the nucleus, NF-κB activity is favored by nuclear thioredoxin-1 (Trx1) and can repair oxidatively damaged proteins. NF-κB activity is inhibited by increased nuclear H_2_O_2_ production. During oxidative stress the presence of H_2_O_2_ leads to the dissociation of the Nrf2/Keap1 complex and cysteinyl residues of Keap1 are modified. Nrf2 translocates into the nucleus to induce expression of its target genes that triggers antioxidant signaling [59].

As mentioned before, H_2_O_2_, acting as a second messenger in the redox metabolism, is able to activate the response of the cells to different stimuli (for example binding of growth factors to their receptors as discussed in Section 3 or apoptotic cell death as discussed in Section 5). The action of H_2_O_2_ as a second messenger molecule also impacts on cell transcription. In fact, redox-sensitive transcription factors such as NF-κB and nuclear factor, erythroid 2 like 2 (NFE2L2 or NRF2) are considered redox signaling targets that can, in turn, regulate many cellular functions. The mechanism is illustrated in Figure 3.

The NF-κB is a regulator factor involved in inflammation, innate and adaptive immune response, viral infection, proliferation and apoptosis [7,60]. The Rel/NF-κB transcription factors are composed of several proteins which contain a Ref-1-homology domain (RHD), that allows dimerization, recognition and binding to DNA. Moreover, the Rel/NF-κB contains a NLS, which interacts with the inhibitory proteins IκBs, regulated and expressed in a tissue specific manner [61,62]. The oxidation by cytosolic H_2_O_2_ leads to the dissociation of the NF-κB/IκB complex, so that the NF-κB can translocate into the nucleus. Furthermore, NF-κB possesses a DNA-binding subunit with redox-sensitive Cys residues: the NF-κB activity is favored by nuclear thioredoxin-1 (Trx1), involved in many cellular processes, including repair of oxidatively damaged proteins, protein folding and redox homeostasis. On the other hand, the NF-κB activity is inhibited by increased nuclear H_2_O_2_ production [59,63,64].

Another important transcription factor is NRF2, a regulator of those genes encoding for antioxidant and detoxifying enzymes in response to oxidative stress. In physiological conditions, Kelch like ECH associated protein 1 (KEAP1) sequesters NRF2 in the cytoplasm: thus, the latter cannot translocate into the nucleus and its transcriptional activity is suppressed. In this condition, NRF2 can be degraded through two pathways: a proteasome-dependent rapid turnover or a slow turnover in the nucleus [65,66]. During oxidative stress the presence of H_2_O_2_ leads to the dissociation of the NRF2/KEAP1 complex and specific cysteinyl residues of KEAP1 are modified. This alteration causes KEAP1 to lose its ability to hold back NRF2: the latter translocates into the nucleus to induce expression of its target genes, triggering antioxidant signaling [59,67].

GR and TrxR are enzymatic systems able to maintain the stability of the NRF2/KEAP1 and NF-κB/IκB complexes. In this regard, the selenoprotein thioredoxin reductase 1 (TrxR1) is considered a potent regulator of NRF2: TrxR1 cooperates with KEAP1 in detecting oxidative stress and modulating appropriate NRF2-dependent responses [59,68].

## 9. H_2_O_2_ in Disease Settings: Ischemia Reperfusion Injury

Among ROS-related diseases, ischemia reperfusion (I/R) injury is one of the most studied conditions associated to myocardial infarction, stroke, and other thrombotic events. Tissue reperfusion injury occurs when blood circulation returns to the tissue after a period of ischemia. The absence of nutrients and oxygen creates a condition in which the restoration of the circulation results in aberrant inflammation and oxidative stress with consequent damage to the tissues involved. Several molecular mechanisms have been proposed to explain I/R injury [69,70]. Several studies, aimed at identifying the link between ROS and I/R injury, reported that accelerated ROS production in post-ischemic tissues is caused by enzymes that are able to reduce molecular oxygen to form superoxide and/or H_2_O_2_, thus releasing H_2_O_2_ in the extra- and intra-cellular spaces [69]. NO and superoxide are involved in altered endothelial-dependent responses. During the I/R injury, the endothelium-dependent vasodilation in arterioles is compromised and the inflammatory response in venules alters the equilibrium between NO and superoxide in endothelial cells. Under physiological conditions the flow of NO exceeds that of superoxide and this situation prevents platelet aggregation and inhibits adhesive interactions between leukocytes and the endothelial cell surface. After the reperfusion of the ischemic tissues, the production of superoxide, which has higher levels than those of NO, is favored. For this reason the endothelial cells produce more superoxide molecules and the decrease of NO from endothelial NO synthase occurs [70,71]. Moreover, NO is not available to act as a second messenger and endothelium-dependent vasodilation is compromised. When superoxide accumulation occurs, the production of H_2_O_2_ is favored and these two metabolites cause rapidly platelet-activating factor production via phospholipase activation and mediate the initial expression of P-selectin by mobilizing the leukocyte rolling receptor from its preformed pool in endothelial cells [70]. Under the ischemic period, the antioxidant defenses are also damaged and the increase in H_2_O_2_ generates the hydroxyl radical which, in turn, causes a direct injury to the cell membranes, proteins and lipids. Following the restoration of oxygen occurring via the reperfusion, the production of ROS by dysfunctional mitochondria increases dramatically. In this regard, xanthine oxidase (XO) further increases ROS production by converting hypoxanthine and O_2_ into highly reactive superoxide (O_2_^−^) [69,72]. The release of ROS in post-ischemic tissues can occur in intracellular or extracellular compartments. Together with XO, the enzyme systems involved in accelerated ROS production are the mitochondrial electron chain and NOX. XO, which belongs to oxidoreductase family, is able to catalyze the oxidations of several substrates such as purines, aldehydes, and heterocyclic molecules. Moreover, some electron acceptors such as O_2_ and nitrate, cooperate with XO. The mammalian form of this enzyme exists in two forms, xanthine dehydrogenase (XDH) and XO. XDH uses NAD+ as an electron acceptor, while XO uses O_2_ as the terminal electron acceptor with the ability to generate ROS [69]. Thus, XO plays an important role in I/R injury. During ischemia, an increase in purines derived from ATP catabolism occurs. The XO is also able to convert xanthine into uric acid. During reperfusion the restored oxygen supply allows the XO to form uric acid starting from the accumulated purines; the by-products of the reaction are H_2_O_2_ and O_2_^−^ (ROS) [73]. At significantly high concentrations (10–100 μM), H_2_O_2_ may cause phenotypic changes in endothelial function that may occur in post-ischemic tissues; these manifestations include endothelial barrier dysfunction (increased vascular permeability), increased adhesion of leukocyte-endothelial cells, increased expression of endothelial cell adhesion molecules, production of inflammatory mediators and the induction of a prothrombotic setting [74,75,76,77].

## 10. H_2_O_2_ and the Protective Signaling Pathways: The Ectonucleotidases

**Figure 4:**
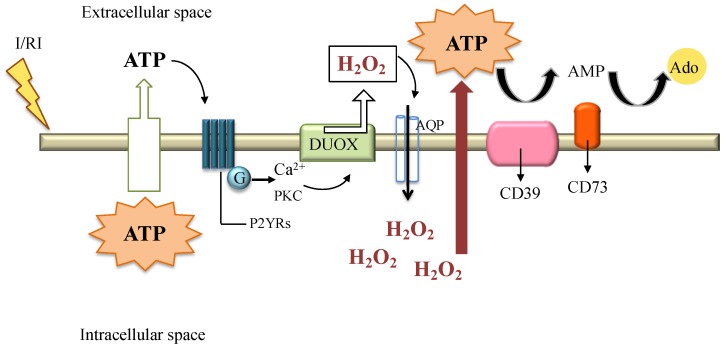
Ischemia reperfusion injury (I/RI) induces the passive or active release of intracellular ATP to the extracellular space. Once in the extracellular milieu, ATP can activate G-protein-coupled receptors (P2YRs) to stimulate calcium-dependent signaling and the activation of protein kinase C (PKC) allowing the activation of the DUOX complex and the release of H_2_O_2_. H_2_O_2_ can cross the cellular membrane via AQP to initiate redox signaling and further promote ATP efflux. Extracellular ATP is metabolized by enzymatic phosphohydrolysis in a two-step process via CD39 conversion of ATP to AMP, and CD73 phosphohydrolysis of AMP to adenosine (Ado). The latter, can mediate anti-inflammatory effects [50,79,84,85].

The detrimental effects of H_2_O_2_ and ROS in ischemic and post-ischemic tissues, together with the absence of oxygen (ischemia) or aberrant inflammatory response (reperfusion), have the consequence to induce several cells in the infarcted area to undergo necrotic or apoptotic cell death. Necrotic cells passively extrude ATP into the extracellular space, while apoptotic cells actively release ATP into the extracellular space via pannexin hemi-channels. Moreover, inflammatory cells that were recruited and activated during reperfusion also release intracellular ATP via connexin hemi-channels [78,79], as illustrated in Figure 4. Once in the extracellular milieu, ATP mediates inflammatory effects upon binding to cell surface type 2 (P2) purinergic receptors (P2XRs) and G-protein-coupled receptors (P2YRs). Elevated concentrations of ATP in the extracellular space activate inflammation in I/R injury via the P2X7 signaling [80]. In addition, purinergic signaling also has a profound impact upon chronic responses, including cell proliferation, differentiation and apoptosis, such as seen in atherosclerosis, neurodegenerative diseases, and in several inflammatory conditions [81,82,83].

The two most important ecto-enzymes that possess anti-inflammatory properties, are involved in the purinergic signaling. ENTPD1/CD39 (ectonucleoside triphosphate diphosphohydrolase-1) is a plasma membrane protein whose expression is regulated by several pro-inflammatory cytokines, oxidative stress and hypoxia through transcription factors such as specificity protein 1 (Sp1) and signal transducer and activator of transcription 3 (Stat3) [86,87,88]. CD39 is the main ectonucleotidase expressed by human and murine Tregs cells. CD39 hydrolyzes extracellular ATP and adenosine diphosphate (ADP) into adenosine monophosphate (AMP). AMP is then processed into immunosuppressive adenosine by the ecto-5′-nucleotidase (E5NT/CD73). CD73 is a glycosyl phosphatidylinositol (GPI)-linked membrane-bound glycoprotein, which hydrolyzes the extracellular nucleoside monophosphates into bioactive nucleoside intermediates. Surface-bound CD73 metabolizes adenosine 5′-monophosphate (AMP) to adenosine, which can activate one of four types of G-protein coupled or can be internalized through dipyridamole-sensitive carriers [89,90]. The enzymatic activity of the CD39/CD73 axis is illustrated in Figure 4. CD73 is important in a series of biological events, such as cell survival, proliferation, and cell motility. There is evidence that the expression and function of this enzyme are upregulated under hypoxic conditions, as well as by the presence of several pro-inflammatory mediators, such as transforming growth factor (TGF)-β, tumor necrosis factor (TNF)-α, interferons (IFNs), and interleukin (IL)-1β [91,92,93,94]. The major function of CD73 is the production of extracellular adenosine from extracellular AMP. CD73-derived adenosine has the ability of suppressing inflammatory reactions and mediating cardioprotective mechanisms and vasodilatation. Adenosine, besides controlling the migration of neutrophils, also regulates the lymphocytes migration through the endothelial cell barrier. It restricts the infiltration of neutrophils into tissues, and thus protects it from inflammatory damage. Therefore, the coordinated action of CD39/CD73 axis results in the rapid conversion of extracellular ATP to adenosine, leading to the activation of a protective change of metabolism of cells, as we reported in a model of endothelial cells exposed to H_2_O_2_ stimulus [95]. This protective metabolic change could have impact on immunological response: adenosine mainly mediates anti-inflammatory effects, while ATP in the extracellular space acts as a strong pro-inflammatory stimulus on T cells, dendritic cells, and neutrophils [85]. An increase of intracellular Ca^2+^ and extracellular ATP are linked to the release of ROS, especially H_2_O_2_, which is produced by the DUOX enzyme system: H_2_O_2_ acts on target cells to trigger the redox signal, reason why it can be considered another cellular damage signal [50].

## 11. H_2_O_2_ and the Protective Signaling Pathways: The Heme Oxygenase-1

H_2_O_2_ can also increase oxidative stress and redox signaling at high concentrations as well as NO and heme. These stimuli can induce the expression of the *Heme Oxygenase-1* (*HMOX1*) gene, encoding for the potent anti-inflammatory protein HO-1, to prevent tissue injury by modulating the immune responses [96,97,98]: HO-1 can be considered as an enzymatic antioxidant [99]. The heme, the substrate of the HO-1 enzymatic reaction, is a pro-oxidant molecule that can participate in the formation of ROS, at the end leading to oxidative injury [100]. Heme is degraded by the rate-limiting enzyme HO-1 that possesses antioxidant function and promotes many biological oxidation processes involved in oxygen transport, mitochondrial respiration, cellular antioxidant defenses, and signal transduction processes. For these reasons heme proteins are considered a source of ROS and their acute exposure aggravates oxidative injury [101,102]. The formation of H_2_O_2_ in erythrocytes is associated with the autoxidation of oxyhemoglobin, a process that produces superoxide, which, in turn, reacts with SOD enzymes producing H_2_O_2_ and oxygen. The presence of hemoglobin, eventually released to the extracellular space from damaged erythrocytes, increases the toxicity of H_2_O_2_. In fact, the oxidation of extracellular hemoglobin induces the release of heme, which is then incorporated into the plasma membrane of endothelial cells where it releases its iron [103]. These pro-oxidant stimuli are sensed by the cell, leading to the activation of the expression of *HMOX1* gene. The binding of heme to ferritin, which is co-induced with HO-1, may prevent oxidative stress. The HO-1 and ferritin synthesis in the endothelium protects it from subsequent exposure to H_2_O_2_ and leads to neutrophils activation [104]. The three products of HO-1 action on heme can mediate these effects: carbon monoxide (CO), biliverdin and free iron (Fe^2+^). Fe^2+^ upregulates an iron-transporter pump that removes Fe^2+^ from the cell and promotes the expression of ferritin. The latter reduces the generation of free radicals by binding Fe^2+^, thus avoiding the Fenton reaction (H_2_O_2_ + Fe^2+^ → Fe^3+^ + OH∙ + OH^−^) that would promote the generation of free radicals. In particular, in the presence of free transition metals (iron), H_2_O_2_ originates the hydroxyl radical (OH^-^), which is toxic and responsible for lipid peroxidation.

The heme degradation catalyzed by HO-1 generates biliverdin, which has potent anti-oxidant effects and is rapidly converted to bilirubin by the action of biliverdin reductase. Bilirubin is a lipophilic and water insoluble compound that can be considered a ROS scavenger and a powerful antioxidant [105]. CO is produced during heme degradation and intracellular CO can influence the activity of other cellular hemoproteins such as cytochrome p-450, NOX, nitric oxide synthase (NOS), and cyt-*c* oxidase, which are involved in important processes such as cellular respiration. CO, as a redox-active heme metabolite, can also participate in cellular defense mechanisms [104]. Another important class of enzymes that detoxify the cell is represented by the CATs that are heme enzymes designed to remove H_2_O_2_ through disproportionation. The CAT contains four iron-containing heme groups that react with the H_2_O_2_. Peroxidases utilize H_2_O_2_ to generate highly oxidized hemes that are then used in substrate oxidation. Several hemoproteins such as cytochrome P450 isoenzymes, cyt-*c* and hemoglobin act as oxidants [106,107].

## 12. Conclusions

H_2_O_2_ is a central player in the redox signaling in cells, where it can contribute to homeostatic metabolism or the toxic response and damages on the basis of cellular localization, concentration, and upstream/downstream interactors. The cells have several systems as source of H_2_O_2_ but, at the same time, they also possess scavenger molecules to strictly control its concentration in different subcellular compartments. The further understanding of which molecules and targets are involved in the delicate balance between the physiological concentration of H_2_O_2_ as a result of homeostatic oxidative stress (referred as ‘oxidative eustress’ by Helmut Sies [3]) and the excessive and pathogenic loading of the same metabolite would clarify what could happen in inflamed tissues and identify novel therapeutic interventions.
